# Report of first recurrent glioma patients examined with PET-MRI prior to re-irradiation

**DOI:** 10.1371/journal.pone.0216111

**Published:** 2019-07-24

**Authors:** Daniel F. Fleischmann, Marcus Unterrainer, Stefanie Corradini, Maya Rottler, Stefan Förster, Christian la Fougère, Timo Siepmann, Markus Schwaiger, Peter Bartenstein, Claus Belka, Nathalie L. Albert, Maximilian Niyazi

**Affiliations:** 1 Department of Radiation Oncology, University Hospital, LMU Munich, Munich, Germany; 2 German Cancer Consortium (DKTK), partner site Munich, Munich, Germany and German Cancer Research Center (DKFZ), Heidelberg, Germany; 3 Center for Clinical Research and Management Education, Division of Health Care Sciences, Dresden International University, Dresden, Germany; 4 Department of Nuclear Medicine, University Hospital, LMU Munich, Munich, Germany; 5 Department of Nuclear Medicine, Klinikum Bayreuth, Bayreuth, Germany; 6 Department of Nuclear Medicine and Clinical Molecular Imaging, University of Tuebingen, Tuebingen, Germany; 7 German Cancer Consortium (DKTK), partner site Tuebingen, Tuebingen, Germany and German Cancer Research Center (DKFZ), Heidelberg, Germany; 8 Department of Neurology, Carl Gustav Carus University Hospital, TU Dresden, Dresden, Germany; 9 Department of Nuclear Medicine, Technical University of Munich, Munich, Germany; Ohio State University, UNITED STATES

## Abstract

**Background and purpose:**

The advantage of combined PET-MRI over sequential PET and MRI is the high spatial conformity and the absence of time delay between the examinations. The benefit of this technique for planning of re-irradiation (re-RT) treatment is unkown yet. Imaging data from a phase 1 trial of re-RT for recurrent glioma was analysed to assess whether planning target volumes and treatment margins in glioma re-RT can be adjusted by PET-MRI with rater independent PET based biological tumour volumes (BTVs).

**Patients and methods:**

Combined PET-MRI with the tracer O-(2-^18^F-fluoroethyl)-l-tyrosine (^18^F-FET) prior to re-RT was performed in recurrent glioma patients in a phase I trial. GTVs including all regions suspicious of tumour on contrast enhanced MRI were delineated by three experienced radiation oncologists and included into MRI based consensus GTVs (_MR_GTVs). BTVs were semi-automatically delineated with a fixed threshold of 1.6 x background activity. Corresponding BTVs and _MR_GTVs were fused into union volume _PET-MR_GTVs. The Sørensen–Dice coefficient and the conformity index were used to assess the geometric overlap of the BTVs with the _MR_GTVs. A recurrence pattern analysis was performed based on the original planning target volumes (PTVs = GTV + 10 mm margin or 5 mm in one case) and the _PET-MR_GTVs with margins of 10, 8, 5 and 3 mm.

**Results:**

Seven recurrent glioma patients, who received PET-MRI prior to re-RT, were included into the present planning study. At the time of re-RT, patients were in median 54 years old and had a median Karnofsky Performance Status (KPS) score of 80. Median post-recurrence survival after the beginning of re-RT was 13 months. Concomitant bevacizumab therapy was applied in six patients and one patient received chemoradiation with temozolomide. Median GTV volumes of the three radiation oncologists were 35.0, 37.5 and 40.5 cubic centimeters (cc) and median _MR_GTV volume 41.8 cc. Median BTV volume was 36.6 cc and median _PET-MR_GTV volume 59.3 cc. The median Sørensen–Dice coefficient for the comparison between _MR_GTV and BTV was 0.61 and the median conformity index 0.44. Recurrence pattern analysis revealed two central, two in-field and one distant recurrence within both, the original PTV, as well as the _PET-MR_GTV with a reduced margin of 3 mm.

**Conclusion:**

PET-MRI provides radiation treatment planning imaging with high spatial and timely conformity for high-grade glioma patients treated with re-RT with potential advancements for target volume delineation. Prospective randomised trials are warranted to further investigate the treatment benefits of PET-MRI based re-RT planning.

## Introduction

Prognosis for patients suffering from high-grade glioma remains poor, with a median expected survival of 42 months for patients with anaplastic astrocytomas and 15 months for patients with histology of glioblastoma [1]. Moreover, recurrent disease is diagnosed in the majority of glioma patients. Re-irradiation (re-RT) has been reported to be an efficient retreatment option [2] whereas neurosurgical re-intervention at the time of recurrence is limited to a selected patient group [3,4].

As most patients initially receive multimodal therapy, the decision to offer re-RT is often complicated by differentiating post-treatment changes from recurrent/progressive tumour, requiring additional information on tumour metabolism of positron emission tomography (PET) imaging [5,6]. PET imaging with amino acids as O-(2-^18^F-fluoroethyl)-l-tyrosine (^18^F-FET) provides valuable information for radiation planning as areas of metabolically active tumour can be differentiated from normal brain tissue [7]. The use of PET with amino acids such as ^18^F-FET for re-RT treatment planning is prospectively examined in the GLIAA/NOA-10 trial and emphasized by the working group on Response Assessment in Neuro-Oncology (RANO) as target volume definition may be improved compared to approaches based on MRI only [8]. The Advisory Committee on Radiation Oncology Practice of the ESTRO similarly recognizes the advantage of ^18^F-FET PET for recurrent glioma re-RT planning, but does not generally recommend the addition of ^18^F-FET PET for primary glioblastoma treatment planning outside of clinical trials [9].

PET-MRI enables acquisition of MR and PET imaging within one diagnostic setting. As patients are placed in the exact same position during both examinations and co-registration of the images is performed on-site, mismatches of the two imaging modalities are greatly reduced. Proper spatial conformity of PET and MRI in the combined PET-MRI examination has been reported to be beneficial for radiotherapy treatment planning [10,11] and might reduce interrater variability [12].

Combined PET-MRI has recently been described as an imaging modality with very high specificity and sensitivity in detecting glioma recurrence [13]. Moreover, the diagnostic confidence in the radiological detection of primary brain tumours has been described to be higher for combined PET-MRI in comparison to MRI alone [14].

The aim of this phase I trial (NCT01579253) was to utilize PET-MRI for re-RT treatment planning of high-grade glioma patients. Re-RT treatment planning data were analysed to assess the additional value of PET-MRI in the re-RT target delineation process. ^18^F-FET PET based biological tumour volumes (BTVs) were compared to MRI based gross tumour volumes (GTVs) of three radiation oncologists. A recurrence pattern analysis was subsequently conducted including the original planning target volumes (PTVs) and volumes containing BTVs. The aim of the recurrence pattern analysis was to assess whether margin adjustment using metabolic ^18^F-FET PET information of PET-MRI could be beneficial on local control.

## Patients and methods

### Patients

Patients with recurrent glioma (WHO grade III or IV) were eligible for the present study, if re-RT was applicable regarding prior radiation treatment, which had to be conducted more than six months prior to re-RT. Enrolled patients had to be between 18 and 75 years old and be able to freely provide informed consent. Claustrophobia, implanted medical devices and metallic objects as well as pregnancy were exclusion criteria for the study to ensure safety at MRI examination. The ethics committee of the LMU Munich reviewed the study protocol and gave approval before the beginning of patient enrollment (study number 361–11). The study was registered at the clinicaltrials.gov registry (NCT01579253). All patients provided written informed consent before study enrollment.

### ^18^F-FET PET-MRI

The phase I clinical trial on combined PET-MRI prior to re-RT of patients with recurrent WHO grade III or IV glioma (NCT01579253) was conducted between 04/2012 and 05/2014 with the tracer O-(2-[^18^F]fluoroethyl)-l-tyrosine (^18^F-FET) at the University Hospital of the Technical University of Munich. As preparation to the ^18^F-FET PET scan, patients had to fast for at least 6 hours before the examination. PET scan started after i.v. injection of ^18^F-FET with a mean activity of 181.7 +- 25.6 MBq and was performed for 40 minutes. Static images were achieved though summation of the images of the timespan 20 until 40 minutes after i.v. injection. The ^18^F-FET PET was acquired on a Siemens mMR Biograph, Knoxville, TN. PET images were reconstructed using a filtered back projection reconstruction algorithm and a 4.9 mm Hann filter with a matrix size of 128, a scaling factor of 2.24 mm/pixel, and a slice thickness of 0.91 pixels. The fl3d1_ns T1 sequence had a matrix size of 256 pixels, a scaling factor of 1 mm/pixel, a slice thickness of 1 pixel, a center-center slice separation of 1 pixel, a TE of 4.76 ms and a TR of 11 ms.

On a HERMES work station (Hermes Medical Solutions, Sweden), the mean ^18^F-FET tracer uptake of the healthy background was evaluated in a crescent-shaped VOI in the contralateral hemisphere, as recently published [15]. Biological tumour volumes (BTVs) were subsequently semi-automatically generated with a fixed threshold SUV_max_/BG ratio of 1.6, as this ratio showed high sensitivity and specificity of tumour tissue detection in a ^18^F-FET PET guided biopsy study [16]. MRI included standard T1 sequences with gadolinium contrast medium and T2 fluid attenuation inversion recovery (FLAIR) sequences. Gadobutrol contrast medium was used at a dose of 0.2 mmol per kg bodyweight. Co-registration of PET and MR images was automatically performed on-site after image acquisition.

### Treatment schedule

Re-RT was performed after consensus decision within the interdisciplinary neuro-oncological tumour board of the University Hospital of Munich as described previously in detail [2]. In summary, patients received 36 Gy total dose in 2 Gy single fractions using 3D conformal or intensity-modulated RT depending on the complexity/shape of the target volume and adjacent critical structures. Thermoplastic masks were used to ensure immobilization of the head during RT. Target volume delineation for re-RT planning was based on the combined PET-MRI, which was co-registered semi-automatically to the planning CT using the treatment planning system Oncentra External Beam (version 4.5, Nucletron, Elekta AB, Sweden). All imaging modalities had a slice thickness of 3 mm. GTV delineation was based on the morphologically suspicious areas on contrast enhanced MRI. PTV delineation included the GTV with a margin of 10 mm and all suspicious areas on ^18^F-FET PET imaging.

Concomitant systemic therapy with bevacizumab at day 1 and day 15 of re-RT was applied at a dosage of 10 mg/kg bodyweight, if no contraindications as artheroembolism, wound healing disorders or arterial hypertension were present in the past medical history. Maintenance therapy of bevacizumab after re-RT was applied after follow-up decision of the neuro-oncological tumour board. Concomitant chemotherapy with temozolomide was applied at re-RT at a dosage of 75 mg/m^2^.

### Comparative and recurrence pattern analysis

**Fig 1:**
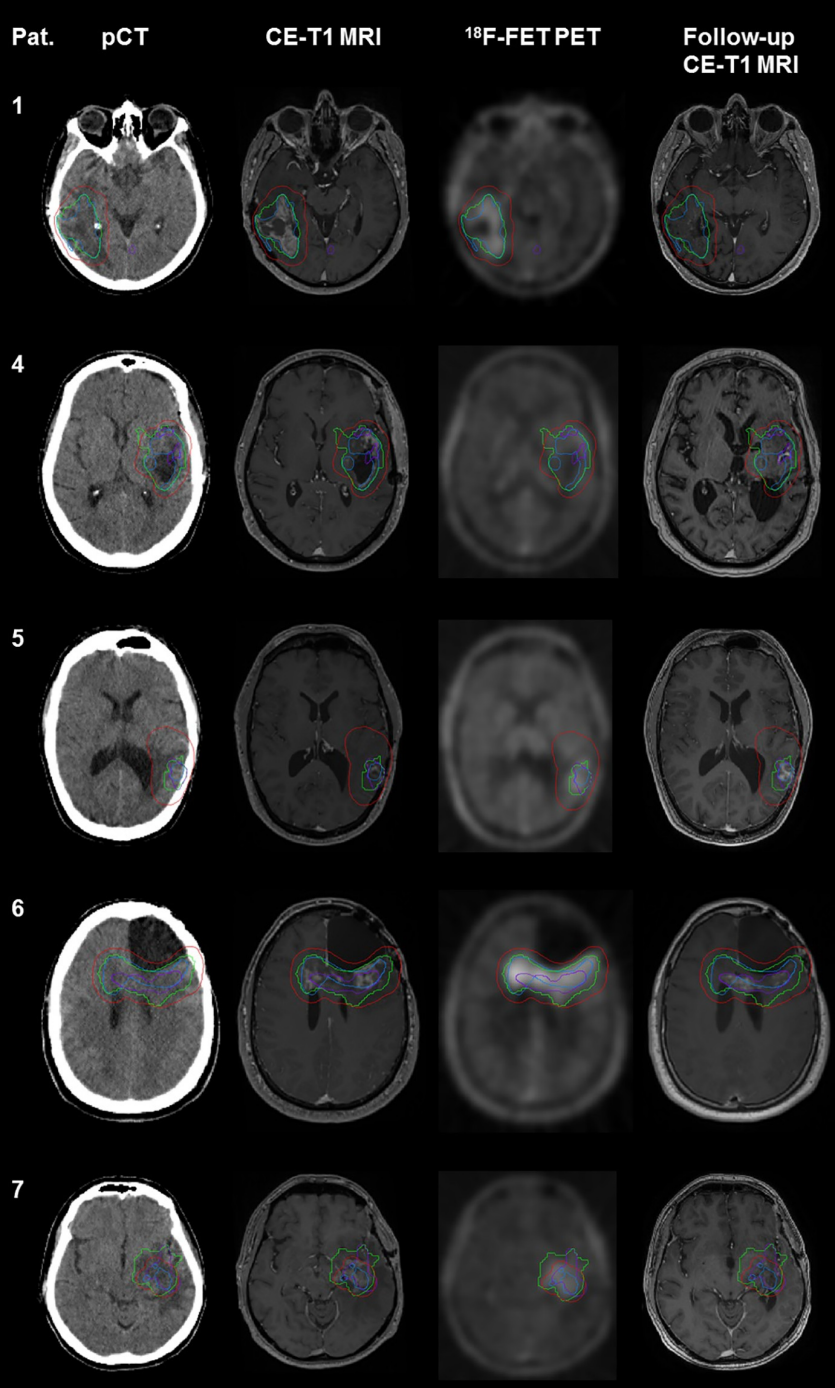
Treatment plan and pattern of recurrence. Original MRI based GTVs in blue, _PET-MR_GTVs in green, CTVs resembling original PTVs minus 3 mm in red and recurrent tumour volumes in follow-up MRI in purple for all patients with available follow-up MRI at recurrence. In patients 1, 4 and 7 no PET information was contained in the original target volumes.

Three independent experienced radiation oncologists delineated the GTV based on the morphologically suspicious findings on MRI. Subsequently, a MRI based consensus GTV (_MR_GTV) was built based on the composite volume of the three GTVs. ^18^F-FET PET based BTVs with a threshold of 1.6 were compared to the _MR_GTVs regarding median size and overlap of the volumes (Fig 1). The volumes of _MR_GTVs and BTVs were fused into a union volume _PET-MR_GTV. A recurrence pattern analysis was performed based on the original PTV (equals GTV with 10 mm margin (n = 6) and GTV with 5 mm margin (n = 1)) and the union structure _PET-MR_GTV with gradually reduced margins from 10, 8, 5 to 3 mm to assess the effects of PET-MRI based target volume and safety margin reduction. Additionally, _PET-MR_GTV was compared to CTVs, which were delineated on basis of the original PTVs with a margin reduction of 3 mm.

The recurrent tumour was delineated in the first follow-up MRI after re-RT displaying tumour re-recurrence and included tumour suspicious contrast-enhancing areas of the T1 sequence. As for two patients, no follow-up MRI at the time of recurrence was available, recurrence pattern analysis could only be performed for five patients. Pseudo-progression was ruled out by further examinations, in n = 4 patients through validation of tumor progression in ^18^F-FET PET imaging and in n = 1 patient through stereotactic biopsy with histopathological diagnosis of vital tumor.

**Fig 2:**
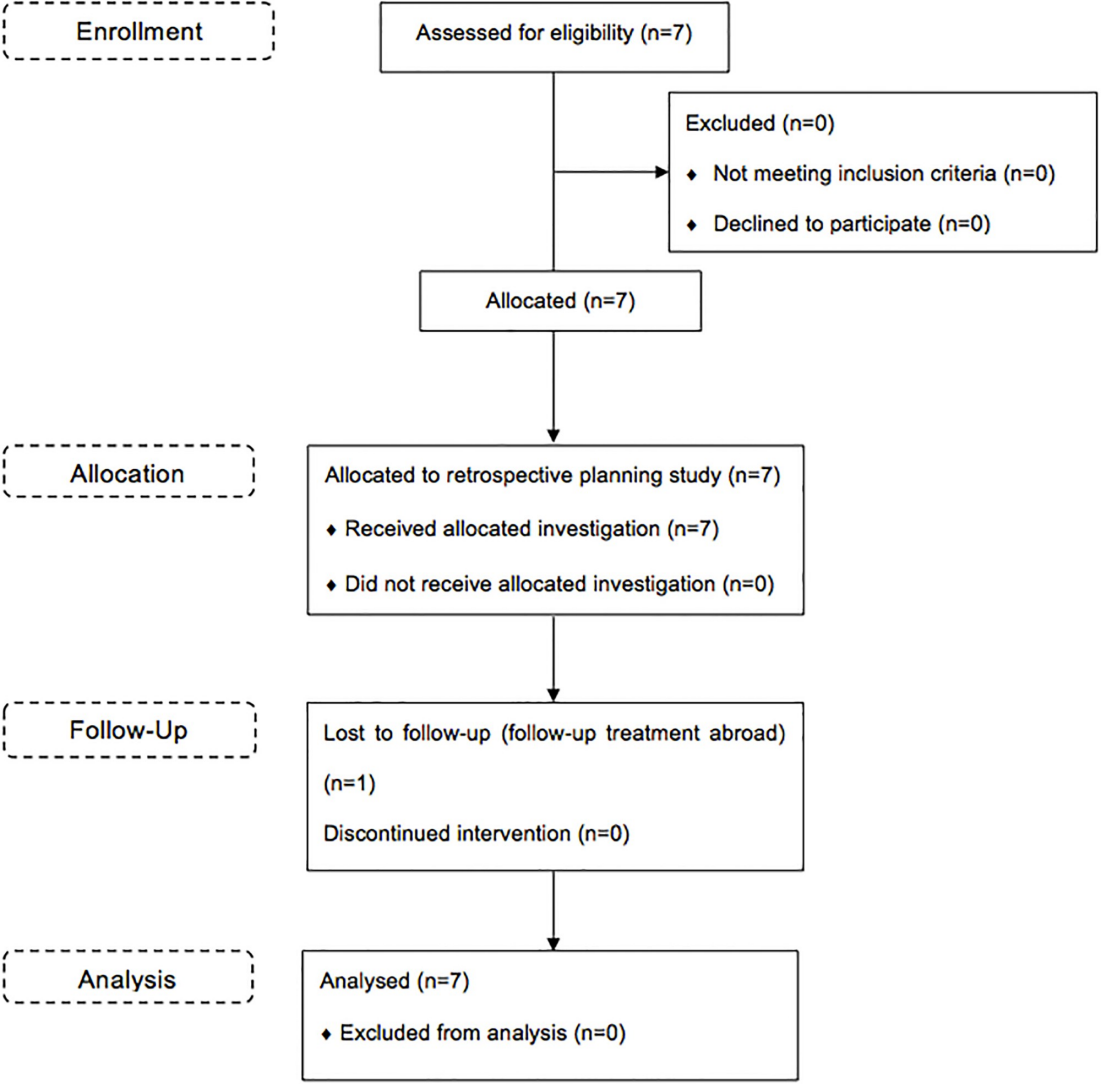
CONSORT flowchart. Flowchart on enrollment, allocation, follow-up and analysis of the retrospective planning study.

The type of recurrence was classified according to the overlap of the recurrent tumour with the respective target volume as central (95–100%), in-field (80–95%), marginal (20–80%) or distant recurrence (0–20%) as defined by Chan et al [17]. A flowchart on the enrollment, allocation, follow-up and analysis of the retrospective planning study is shown in Fig 2.

### Statistical data analysis

Descriptive statistics were used for patient characteristics, histopathological classification, treatment regimens and tumour volumes. Kaplan-Meier estimators were used for survival analysis. The time duration from initial diagnosis of the primary tumour until death or last follow-up was defined as overall survival and from initial diagnosis of the primary tumour until beginning of re-RT as progression-free survival (PFS). The time duration from beginning of re-RT until death or last follow-up was defined as post-recurrence survival (PRS) and from beginning of re-RT until first follow-up MRI showing tumour re-recurrence as post-recurrence progression-free survival (PR-PFS). Two-way random average measures intraclass correlation coefficient analysis was performed to assess the concordance between the GTV delineation of the three raters. The overlap of volumes of the _MR_GTVs and the corresponding BTV was calculated using the Sørensen–Dice coefficient [18] and the conformity index [19]. The Sørensen–Dice coefficient was calculated with the formula 2 x |_MR_GTV∩BTV| / (|_MR_GTV| + |BTV|) and the conformity index with the formula |_MR_GTV∩BTV| / |_MR_GTV∪BTV|. Statistical analysis was performed with the Statistical Package for Social Sciences (SPSS, Version 24.0, IBM SPSS Statistics, Armonk, NY, USA).

### Ethical

Written informed consent was provided by all patients before entering the study.

## Results

### Patients

Seven male patients with recurrent glioma as diagnosed on MRI or by stereotactical biopsy with subsequent histopathological examination were included into the present analysis. Five patients suffered from recurrent WHO grade IV glioblastomas and two patients from WHO grade III anaplastic astrocytomas. MGMT promotor methylation status was available for all patients, which showed a methylated MGMT promotor in three patients (42.9%). IDH1/2 mutational status was only available for 6 patients showing mutation of the IDH1 gene in one patient (14.3%) and no mutation of the IDH2 gene. Primary treatment is listed in detail in Table 1.

**Table 1:** Patient and treatment characteristics. Progression-free survival (PFS); re-treatment, post-recurrence survival (PRS) and post-recurrence progression-free survival (PR-PFS); GB—glioblastoma, AA—anaplastic astrocytoma, GTR—gross total resection, STR—subtotal resection, RT—radiotherapy, RCX—radiochemotherapy, BEV—bevacizumab, TMZ—temozolomide, PDT—photodynamic therapy.

Pt	Patient Characteristics	Primary Treatment	PFS [months]	Re-Treatment	PR-PFS [months]	PRS [months]
1	62 y, male, GB, right temporal lobe, MGMT unmethylated, IDH1-wildtype	GTR, RCX with TMZ, adj. TMZ	11	re-RT with BEV, BEV maintenance	7	13
2	47 y, male, AA, left frontal lobe, MGMT methylated, IDH1-wildtype	STR, RCX with TMZ, adj. TMZ	40	re-RT with BEV	0	0 (lost to follow up)
3	69 y, male, GB, left frontal lobe, MGMT unmethylated, IDH1-wildtype	Biopsy, PDT, RCX with TMZ, adj. TMZ	14	re-RT with BEV, BEV maintenance	2	2
4	54 y, male, GB, left temporal lobe, MGMT methylated, IDH1-wildtype	Biopsy, RCX with TMZ, adj. TMZ	9	STR, re-RT with BEV, BEV maintenance	8	33
5	46 y, male, GB, left parietal lobe, MGMT unmethylated, IDH1-wildtype	GTR, RCX with TMZ, adj. TMZ, Seeds, PDT	36	re-RT with TMZ, Seeds	8	26
6	34 y, male, AA, left frontal lobe, MGMT methylated, IDH1 mutated	GTR, RCX with ACNU/Teniposid, STR, adj. TMZ, Seeds, TMZ	125	re-RT with BEV, BEV maintenance, TMZ, Novo-TTF	10	25
7	61 y, male, GB, left temporal lobe, MGMT unmethylated, IDH1-wildtype	GTR, RT	10	re-RT with BEV	3	5

All patients received combined PET-MR imaging prior to re-RT. Regarding tumour localisation, all recurrent tumours were located in the same lobe as the primary tumour, three of which were located in the frontal (42.9%), one in the parietal (14.2%) and three in the temporal lobe (42.9%).

All patients were treated with re-RT at recurrence. Median age at the time of re-RT was 54 years (range 34–69 years). Median KPS at beginning of re-RT was 80 (range 50–100). Bevacizumab was concomitantly applied in 6 patients (85.7%). One patient (14.3%) received temozolomide at recurrence because of a contraindication for bevacizumab therapy. Bevacizumab maintenance therapy was applied in four patients after re-RT with concomitant bevacizumab (57.1%).

Median follow-up time from the initial treatment until death or last follow-up was 52 months. The median overall survival was 43 months for all patients. Median PFS from initial treatment to re-RT treatment was 14 months. Median PRS after beginning of re-RT was 13 months. Median PR-PFS after beginning of re-RT until first diagnosis of progression on follow-up MRI was 7 months. One patient was lost to follow-up and in one patient no follow-up MRI was available, due to fast progression of the tumour after re-RT treatment.

### Tumour volumes

The median volume of the GTV was 35.0 cubic centimeters (cc, range 16.5–56.8 cc) for radiation oncologist 1, 40.5 cc (range 13.9–63.8 cc) for radiation oncologist 2 and 37.5 cc (range 19.5–66.0 cc) for radiation oncologist 3. In the intraclass correlation coefficient (ICC) analysis, the absolute agreement between the three raters was on average measures 0.98 (p < 0.001). The median volume of the _MR_GTVs of all three raters was 41.8 cc (range 21.8–70.5 cc). The median volume of the BTV was 36.6 cc (range 11.1–77.8 cc) and the median volume of the _PET-MR_GTV in median 59.3 cc (range 26.1–85.6 cc).

The Sørensen–Dice coefficient comparing the _MR_GTV with the BTVs was in median 0.61 (range 0.16–0.69) and the conformity index in median 0.44 (range 0.08–0.53) (Table 2).

**Table 2:** Tumour volumes and planning target volumes. Biological tumour volume (BTV), gross tumour volume (GTV) of three radiation oncologists and consensus structure (_MR_GTV), union of biological tumour volume with consensus gross tumour volume (_PET-MR_GTV) and corresponding volume with 3 mm margin, original GTV, original planning target volume (PTV) and an extracted clinical target volume (CTV).

Pt	BTV [cc]	GTV1 [cc]	GTV2 [cc]	GTV3 [cc]	_MR_GTV [cc]	_PET-MR_GTV [cc]	_PET-MR_GTV 3 mm [cc]	Orig. GTV [cc]	Orig. PTV [cc]	CTV [cc]
1	43.8	35.0	41.0	40.1	41.8	59.3	91.3	50.0	177.7	127.4
2	36.6	56.8	63.8	66.0	70.5	85.6	130.0	100.8*	200.6^#^	142.8
3	30.3	18.7	26.4	25.1	26.2	37.1	60.0	30.8	129.4	91.1
4	18.3	23.9	13.9	29.2	30.6	45.7	83.0	9.4	89.1	57.0
5	11.1	16.5	20.6	19.5	21.8	26.1	45.8	48.2*	177.9	129.4
6	77.8	37.1	40.5	37.5	46.3	84.3	134.3	61.8*	244.0	178.5
7	65.4	45.0	42.5	43.8	47.0	73.4	117.2	8.0	83.5	50.9
Median	36.6	35.0	40.5	37.5	41.8	59.3	91.3	48.2	177.7	127.4

* PET information included into the original GTV.

^**#**^ 5 mm GTV to PTV margin only

### Recurrence pattern analysis

The recurrence pattern analysis showed three central, one in-field and one distant recurrence within both, the original PTV and the _PET-MR_GTV with margins of 10, 8, 5 and 3 mm. Marginal recurrences were detected neither for PTV nor _PET-MR_GTV with 10, 8, 5 or 3 mm margins. For two patients, no information on the pattern of recurrence was available, since follow-up was performed abroad in patient number 2 and early progression and death without new imaging occurred in patient number 3. Median volume of the original PTV was 177.7 cc (range 83.5–244.0 cc). The median volume of the _PET-MR_GTV with a margin of 3 mm was 91.3 cc (range 45.8–134.3) (Table 3).

**Table 3:** Recurrence pattern analysis. Recurrence pattern in relation to original planning target volume, clinical target volume (original PTV minus 3 mm margin) and union of biological tumour volume with consensus gross tumour volume with a margins of 10, 8, 5 and 3 mm and without a margin; percentage of overlap with the volume of tumour recurrence in brackets.

Pt	Original PTV	CTV (Original PTV minus 3 mm)	_PET-MR_GTV plus 10 mm	_PET-MR_GTV plus 8 mm	_PET-MR_GTV plus 5 mm	_PET-MR_GTV plus 3 mm	_PET-MR_GTV
1	distant (0)	distant (0)	distant (0)	distant (0)	distant (0)	distant (0)	distant (0)
2	-	-	-	-	-	-	-
3	-	-	-	-	-	-	-
4	in-field (0.91)	in-field (0.88)	central (0.98)	central (0.98)	in-field (0.91)	in-field (0.90)	marginal (0.72)
5	central (0.99)	central (0.99)	central (0.99)	central (0.99)	central (0.99)	central (0.99)	central (0.97)
6	central (0.99)	central (0.98)	central (0.99)	central (0.99)	central (0.98)	central (0.98)	central (0.95)
7	in-field (0.84)	marginal (0.76)	in-field (0.93)	in-field (0.90)	in-field (0.87)	in-field (0.84)	marginal (0.75)

## Discussion

Radiation treatment planning in recurrent glioma depends on high-resolution contrast enhanced MR imaging of good quality as addition to planning CT imaging. PET imaging with amino acid tracers such as ^18^F-FET has a high sensitivity for the detection of high-grade glioma [20,21] and provides valuable information about the metabolic activity of the tumour reaching beyond contrast-enhancing areas on MRI [22]. Therefore ^18^F-FET PET is used to complement morphologic MR imaging in radiation treatment planning of glioma [23]. Recently a prospective clinical trial was launched to evaluate the potential benefit of target volume delineation of recurrent glioma of ^18^F-FET PET based versus conventional contrast enhanced MRI based radiation treatment planning [24].

Since target volume delineation based on metabolic FET imaging is always dependent on morphologic MR and planning CT imaging, recurrent glioma patients have to undergo three different imaging examinations [25]. Considerable logistic efforts are therefore required both from mostly heavily disabled patients and also from radiation oncologists, neuroradiologists and nuclear medicine specialists. PET-MRI provides the promising advantage of combining the high-resolution morphologic MR imaging with metabolic FET imaging in one examination, which effectively lowers the logistic efforts. PET-MRI also facilitates an optimal spatial conformity of both imaging modalities as positioning of the patient does not change during the examination and image co-registration is performed on-site [10,26,25]. Potential inaccuracies in image co-registration with the planning CT images are therefore reduced with imaging derived by PET-MR. Another benefit of PET-MRI is the simultaneous acquisition of PET and MR images, which differ from days to sometimes weeks in clinical practice and therefore limit the comparability of the imaging modalities. Despite the advantages of PET-MRI for radiation treatment planning especially for recurrent glioma patients, thus far it has not been evaluated, whether target volume delineation can be improved on the basis of PET-MRI in the re-RT setting.

In the present study the potential benefit of PET-MRI on target volume delineation in radiation treatment planning of recurrent glioma patients was evaluated in a recurrence pattern analysis with the integration of semi-automatically defined PET based biological tumour volumes with a fixed threshold.

The comparative analysis included the original target volumes, which were conventionally delineated on the basis of MR based GTVs with a safety margin of 5 to 10 mm and subsequent margin adjustments based on the ^18^F-FET PET images. In three cases PET information was already included into the GTV volumes. ^18^F-FET PET based biological tumour volumes consisting of a fixed threshold were unified with MR based GTVs and a margin reduction from 10 to 3 mm was performed. There was no change in the pattern of recurrence, when comparing the original PTVs with the union structure _PET-MR_GTV with a reduced safety margin of 3 mm. The median volume of the _PET-MR_GTV with a margin of 3 mm was 91.3 cc and therefore almost half the size of the median original PTV volume of 177.7 cc. When comparing the CTV volume, which was extracted by shrinking the original PTV by a negative margin of 3 mm with the _PET-MR_GTV without a margin, marginal recurrences were seen both of these volumes, which implies, that these volumes alone would be too small as treatment volumes. Challenging patient recruitment within a highly-selected patient cohort resulted in a small sample size of seven patients, which thereby limited the possibilities of statistical testing.

While the MRI based delineation of the GTVs by three independent radiation oncologists showed a high level of concordance on ICC analysis, there were significant differences in the volume overlap between the consensus GTVs and the ^18^F-FET based BTVs, which were in median comparable in size and volume to the corresponding GTVs. In line with previously published studies, the spacial correlation between GTVs and the ^18^F-FET based BTVs was low, which underlies the importance of the additional PET information to merely morphological MR imaging for re-RT treatment planning for recurrent glioma patients [27,28].

For imaging of brain tumours, a PET-MRI with the tracer ^18^F-FET is particularly beneficial, since small lesions may only be detected by increased uptake of the amino acid tracer, which is complemented by high-resolution morphological MRI with optimal spatial conformity in one examination [10]. ^18^F-FET derived BTVs with different thresholds showed high prognostic significance—both for overall survival and progression-free survival after primary radiotherapy of glioblastoma patients in a recently published retrospective study on 146 patients [29]. BTVs also showed prognostic significance for survival after re-RT in a prospective phase I study on 31 patients [30].

In four cases the original GTV was based on the MRI performed prior to PET-MRI, in which the tumour recurrence was diagnosed and PET-MRI was used for control of the target volumes only. This was due to logistic reasons, as it was of highest importance to limit the time delay between MRI diagnosis of recurrence and re-RT treatment. When comparing these original GTV volumes with the GTV volumes contoured for the recurrence pattern analysis on basis of the PET-MRI, the major importance of timely proximity of the treatment planning imaging becomes apparent, as the size of these volumes differs greatly in two cases. Therefore, in the setting of two separate scans with a usual time delay of seven to ten days, the comparability of tumour volumes delineated on this basis, is by default lower than in the setting of combined PET-MRI. In accordance to previous recurrence pattern analyses after re-RT of recurrent glioma [31,32], the predominant location for recurrence in the present study was found within the high-dose regions of the original PTV and the _PET-MR_GTV plus 3 mm margin. Since (re-)recurrences are frequently located within the high-dose regions after initial [17,27] and re-RT [31,32] it seems obvious, that current RT dose concepts using conventionally fractionated 60 Gy at primary RT and 36 Gy at re-RT are insufficient for long-term tumour control. Therefore, it would be very promising to prospectively assess whether local tumour control after re-RT could be enhanced through improved target delineation with reduction of PTV and a local dose escalation using an integrated boost concept based on PET-MR tumour metabolism information. This strategy has frequently been assessed in the past without a specific focus on combined PET-MRI [33–35].

## Conclusions

PET-MRI with spatial and temporal conformity of metabolic ^18^F-FET PET and morphologic MR imaging showed promising results for the optimization of target volume delineation in radiation treatment planning for patients with recurrent glioma. Rater independent ^18^F-FET PET based BTVs, which differed significantly from MR based GTVs in the present study, may be used as a complementing element in target volume delineation. Prospective trials are needed to assess the treatment benefits of PET-MRI based re-RT treatment planning for patients with recurrent glioma.
